# Drugs

**Published:** 1906-01-20

**Authors:** Donald J. Mackintosh

**Affiliations:** Medical Superintendent, Western Infirmary, Glasgow


### HOSPITAL ADMINISTRATION.

### CONSTRUCTION AND ECONOMICS.

### DRUGS.

By DONALD J. MACKINTOSH, M.B., M.V.O., F.R.S.E., Medical Superintendent, Western Infirmary, Glasgow.

IN a somewhat comprehensive paper on "The Control of Hospital Expenditure with Efficiency," which I read before the Hospitals Association in March last, I made brief allusion to the question of expenditure in the Dispensary Department. My remarks were summarised under the query which I propounded. Is it more economical for a hospital to manufacture everything required for this department, etc., or to purchase many of the important galenicals from wholesale experts? and I venture to suggest that we have not yet had any satisfactory reply.

My reference to the subject seems to have stimulated in various quarters that spirit of inquiry through which alone a satisfactory result can be attained. Notes touching on some aspects of the question have appeared in more than one journal, and a more elaborate article from the chief dispenser of a large hospital was recently published in the columns of THE HOSPITAL. In none of these contributions, however, can I find the necessary data which would be of practical value for the guidance of hospital managers.

In making comparisons we must in the first place assume that the finished products will be of standard value, and that whether galenicals be made or purchased, nothing will be accepted to which exception can be taken as regards quality or standard. One recent correspondent in THE HOSPITAL states that when a dispenser says Inf. Calumba. Conc. can be made for 3s. a gallon, he can draw his own conclusions.

The first conclusion I would draw is that the 4s. worth of spirit required to preserve it is not there. Hence we must insist that the finished product, in comparing cost, is in every case up to the requisite standard.

In the second place, as I pointed out in my paper, we must compare *all* the details, as there falls to be considered the expert labour required, the space, the plant, the motive power, etc. In fixing the cost the manufacturer takes all those items into his calculations; and surely they must also be considered by the manufacturing hospital before one can have any sound basis for comparison.

Having thus laid down what appear to be reasonable conditions let me review some of the aspects of the subject as they present themselves.

*Time.*—One of the leading arguments put forward in favour of preparing everything on the premises is the utilisation of spare time. "A Chief Dispenser" cites the case of a hospital of 600 beds, and he indicates that the dispensary staff of such an institution is only fully occupied on two or even three days per week. Now if this hospital has an out-patient department open daily, the spare time must necessarily be reduced thereby to a minimum. At the London Hospital, which, according to report, "has the finest dispensing department of any hospital" (*Chemist and Druggist*, January 30, 1904, p. 186), the out-patient department deals with 169,020 patients as compared with 13,364 in-patients yearly, a proportion of over twelve to one.

The outdoor department must, therefore, absorb much more time than the indoor. If such institutions with corresponding out-patient departments are not over-staffed, the "spare" time must be a negligible quantity, and if efficiency and economy are to go hand in hand there cannot be any "spare time." To make comparisons, therefore, it is first of all necessary to know exactly the amount of work to be overtaken in the dispensary department each day, whether there is an out-patient department, and what it amounts to in numbers, and the number of the staff employed in this department. In the Western Infirmary of Glasgow, we had last year an average of 444 beds fully occupied, and 24,577 new patients, and 110,157 consultations at the out-patient department (which has a staff of three dispensers and a porter). The forenoon of every day is occupied with attention to the supply of medicines, etc., to the wards, and the afternoon of every day, except Sunday, is mainly occupied in dispensing for the out-patient department. I find that last year the total amount paid to manufacturing firms for galenicals was £160. (This does not indicate the total value of galenicals used.) If this £160 worth of galenicals had been made on the premises we could not hope to reduce their cost more than, say, 25 per cent. How far would this saving go to pay wages for the increased work, as at present the staff are fully occupied. The *first* duty of a dispenser is to dispense. I have never claimed that the *only* duty of a pharmacist is to dispense (*Chemist and Druggist*, March 25, p. 465), for I stated in my paper that "there are articles, such as chemical syrups, compound powders, liniments, ointments, suppositories, etc., which can be easily made in every large hospital dispensary, and which are made in the Scotch hospitals."

If the amount paid in any year for purchased galenicals exceeded the amount that would be saved by their manufacture in the hospital without addition to the staff or the installation of expensive plant, I would say make everything on the premises. But if additional salaries are incurred or expensive plant required, then these must surely be taken into the calculation. This, however, leads me to my next point.

*Expert Labour.*—When the question of manufacture *versus* purchase is considered we must at once distinguish between articles which require expert supervision and those which do not. Any hospital porter, for example, can make all the aerated waters required, and it is not necessary to have the constant attention of a skilled pharmacist for the preparation of most of the common ointments or syrups. But when we come to deal with the delicate processes which are involved in, say, the standardisation of preparations of opium and of nux vomica, the position is entirely changed. If you are to have an expert who will devote his attention for the time which the standardising of, say, nux vomica requires, then other considerations than the cost pound for pound come into the question.

In a paper read before the Western Chemists' Association, Mr. Peter MacEwan, F.C.S. (see *Chemist and Druggist*, March 18) states: "The perfection of pharmacopœial processes . . . has forced out of the apprentice's hands many operations entrusted to him once on a time. . . . The law requires that the medicines served to the public in some thousands of pharmacies shall be of certain standard, and competition makes it impossible for each one of the thousands to do this himself and sell at a profit; consequently the thousands go back to the profitable basis, and a hundred or two skilled pharmacists do the work for the whole lot."

We live in an age of specialism, even in pharmacy, and the assaying of drugs is now carried out to an extent which was undreamt of a quarter of a century ago. Preparations are now standardised, as has been pointed out by one of your correspondents, chemically and physiologically, and first-class manufacturing firms are now equipped with elaborate chemical and biological laboratories where finished products can be tested. The work of such pharmaceutical experts has resulted in the production of reliable preparations, constant in their action, independent of the variable amount of active principle, be it alkaloid, glucoside, or resin, which the crude material may contain. Surely these manufacturers can be trusted to identify much more satisfactorily than any individual hospital buyer, say the selected species of strophanthus, to select the choicest crops of selected plant farms, to give the best assay of hyoscyamus. Where can any individual pharmacist, however well qualified theoretically, have the same opportunity for selecting and identifying drugs as is begotten of a life-long experience. The materia medica of the books is *not* the materia medica of Mincing Lane, and the average pharmacist is out of the running as a drug buyer when compared with those experts who practically do nothing else. How is it possible for the hospital pharmacist to devote the time and attention necessary to compete with those experts, without leaving his own legitimate work to be performed by others.

It is quite evident that if a hospital is to manufacture everything required in the dispensary department it must have a staff of experts to carry on this particular part of the work. These delicate operations of standardising (the principle of which was adopted in the latest Pharmacopœia) cannot be safely left in the hands of learners, as has been suggested, if the finished product is to be relied upon.

My argument then resolves itself into this. To get the best and most reliable preparation skilled or specialised labour is esential. Can a hospital of 600 beds be expected to pay for this specialised labour for the output it has, on the same economical basis as the firm whose output is many times this amount? In my opinion the cost of this specialised labour negatives its profitable use.

*The next point is the Plant.*—Here let me quote from the letter of "Ph.C., F.C.S." in The Hospital of April 1, p. 21: "For over thirty years I was pharmacist at two of our large London hospitals. . . . My instincts were always in favour of making my own preparations up to a certain point, that point being so long as it did not involve the purchase and employment of costly machinery and specialised labour. My early experience provided me with a most valuable lesson. An expensive laboratory was built, costing over £1,000, and it never earned the institution one penny, as its maintenance was far more costly than its earning power. Probably if the machinery could have been kept working from morning to night it would have paid its way; but such was not the case, nor was it possible to so employ it. I do not hesitate to say that a plant costing £50 would have sufficed for all the work we did. My statement is confirmed by the fact that on taking up another appointment, where the plant was of a most simple character, and no laboratory existed, we actually made a larger variety of galenicals than were being made at the larger laboratory."

It has been argued that because steam is already provided in a hospital for heating purposes it can be utilised for the economical preparation of galenicals in the dispensary department. Steam ought to be supplied to every hospital dispensary, whether all its preparations are manufactured on the premises or otherwise. But I might point out a fact which is apparently overlooked by those who employ this argument, that the amount of steam required for steam-pans, etc., is trifling in cost as compared with that required for running a drug mill, etc., with its electric motor. So that not only must we calculate the initial cost of the plant, which in these days of rapid progress may be out of date within a few years, but we have the cost of its upkeep.

*Guarantee.*—The question of guarantee has been referred to in the article by "A Chief Dispenser." He has practically assumed that the guarantee of a wholesale house is of little value, but has given no facts to bear this out. The correspondent in The Hospital of November 25 has already dealt with the theory that "because one firm was dishonest all must necessarily be so." In the end the therapeutical test is the final one, and if the products do not come up to what is expected of them, the physician will very soon find this out. This argument is illustrated in a paper by Dr. W. E. Dixon, read before the Therapeutical Society on November 28, 1905, from which I extract the following: "One case was where a patient was being treated for cardiac failure with digitalis without any response; a supply of bio-chemically standardised tincture was then used, with the result that the condition of the patient improved after two or three doses." We must, however, guard against being carried away by the tendency of the age to trust entirely to the products of special firms, as this tends to decoy the medical profession from the Pharmacopœia. It has been ably pointed out by Edmund White, B.Sc., etc., in an address he delivered on November 21 to the Midland Pharmaceutical Association that the principle of standardisation may be carried too far. Every man is entitled to his opinion, but in view of the fact that many men in the front rank of pharmacy purchase their galenicals from wholesale firms, we may take it there *are* firms whose guarantee of purity and standard is not so valueless as has been suggested.

In the paper above referred to Dr. Dixon states: "The lesson to be drawn from this is that retail chemists should only prepare small quantities of tinctures at a time, so as to have them fresh, or buy standardised preparations from wholesalers."

*Education.*—In dealing with the question of education we must first of all consider to what extent the funds of a hospital can be legitimately used for the purpose of training young pharmacists. I find that in some hospitals, besides the installation of expensive plant, a class-room is provided: in one case to accommodate 16 students, and in this room the chief dispenser teaches them to "write their own prescriptions and afterwards dispense them." In the Western Infirmary we have no pharmacy students; the University of Glasgow, which adjoins the grounds of the Western Infirmary, has provided a laboratory for the teaching of pharmacy to medical students, and at present new laboratories are being built by the university authorities in their grounds alongside the Western Infirmary, not only for the teaching of pharmacy to medical students, but a complete laboratory is being provided for the teaching of students of pharmacy. Is it part of the legitimate work of a hospital to provide the space, plant, apparatus, etc., for the teaching of pharmacy?

The recommendation in the report of Sir Edward Fry's Committee of the King's Hospital Fund is sufficiently explicit on this point, when it says: "For the future, the distinction between the hospital and the school should in every case be drawn not only definitely and exactly, but with such clearness that it may be understood by the general public, and so that no question may arise as to the destination and application of moneys contributed, whether by the King's Fund or from any other source."

In order to arrive at a satisfactory conclusion, the drugs and surgical dressings supplied to the out-patient department must be kept separate and distinct from those supplied to the hospital.

If it can be proved that the dispensary department can be more economically worked by purchasing the more complex galenicals from reliable wholesale experts; if it is shown that the medical staff—who are after all the real arbiters in the matter—are satisfied that the products supplied are reliable and uniform in their results, then surely it must seem questionable policy to employ additional expert staff, to install expensive plant, and occupy the valuable space which is so precious in many of our hospital sites.

The suggestion has been made that in order to reduce the large expenditure in hospitals the co-operative principle of purchase or manufacture might be adopted. To many of the smaller institutions this would certainly be of great advantage, and to many of the larger ones it might possibly be a considerable saving. But this question must be left to be decided on its own merits.

